# Biosensor and Lab-on-a-chip Biomarker-identifying Technologies for Oral and Periodontal Diseases

**DOI:** 10.3389/fphar.2020.588480

**Published:** 2020-11-09

**Authors:** Larissa Steigmann, Shogo Maekawa, Corneliu Sima, Suncica Travan, Chin-Wei Wang, William V. Giannobile

**Affiliations:** ^1^ Department of Periodontics and Oral Medicine, School of Dentistry, University of Michigan, Ann Arbor, MI, United States; ^2^ Department of Periodontology, Graduate School of Medical and Dental Sciences, Tokyo Medical and Dental University, Tokyo, Japan; ^3^ Department of Oral Medicine, Infection, and Immunity, Harvard School of Dental Medicine, Boston, MA, United States; ^4^ Biointerfaces Institute and Department of Biomedical Engineering, College of Engineering, University of Michigan, Ann Arbor, MI, United States

**Keywords:** periodontal diseases/periodontitis, patient stratification, precision medicine, biotechnology, saliva, biomarkers

## Abstract

Periodontitis is a complex multifactorial disease that can lead to destruction of tooth supporting tissues and subsequent tooth loss. The most recent global burden of disease studies highlight that severe periodontitis is one of the most prevalent chronic inflammatory conditions affecting humans. Periodontitis risk is attributed to genetics, host-microbiome and environmental factors. Empirical diagnostic and prognostic systems have yet to be validated in the field of periodontics. Early diagnosis and intervention prevents periodontitis progression in most patients. Increased susceptibility and suboptimal control of modifiable risk factors can result in poor response to therapy, and relapse. The chronic immune-inflammatory response to microbial biofilms at the tooth or dental implant surface is associated with systemic conditions such as cardiovascular disease, diabetes or gastrointestinal diseases. Oral fluid-based biomarkers have demonstrated easy accessibility and potential as diagnostics for oral and systemic diseases, including the identification of SARS-CoV-2 in saliva. Advances in biotechnology have led to innovations in lab-on-a-chip and biosensors to interface with oral-based biomarker assessment. This review highlights new developments in oral biomarker discovery and their validation for clinical application to advance precision oral medicine through improved diagnosis, prognosis and patient stratification. Their potential to improve clinical outcomes of periodontitis and associated chronic conditions will benefit the dental and overall public health.

## Introduction - Periodontal Diseases

Periodontitis is one of the most prevalent chronic inflammatory diseases (Kassebaum et al., 2014). An estimated 42% of United States adults have been diagnosed with periodontitis with around 8% being severely affected (Eke et al., 2018). The clinical manifestation includes the loss of supporting tooth structures such as alveolar bone, soft tissue and the periodontal ligament (PDL). The pathogenesis is triggered by an immunoinflammatory response of the host to a bacterial challenge (Kornman et al., 2017). Gingivitis is a reversible periodontal disease that manifests clinically as gingival inflammation without loss of bone or PDL. In susceptible individuals, gingivitis can lead to periodontitis when untreated. Gingivitis always precedes but not always leads to periodontitis. The transition from a controlled inflammatory state to tissue destructive inflammation requires both a dysbiotic oral microbiome and a susceptible host (Hajishengallis et al., 2012; Hajishengallis, 2014). Besides the infectious nature of opportunistic pathogenic bacteria and their byproducts, host genetics governing the immune-inflammatory response as well as behavioral and environmental risk factors play a critical role in the initiation and progression of periodontal diseases (Page and Kornman, 2000). Therefore, the likelihood and rate of progression relies on multiple patient-related factors and cannot be generalized (Tonetti et al., 2018). Studies have shown that independent of external components, disease progression in different populations occurs in dynamic orders with similar rates (Loe et al., 1978; Teles et al., 2016). Periodontitis is an oscillating condition with periods of disease progression, inactivity, and/or regression (Goodson et al., 1982; Teles et al., 2018).

Notable with chronic diseases, early diagnosis is key to long-term treatment success. Classification criteria in the periodontal field have recently been updated (Papapanou et al., 2018) and include the categorization regarding the severity and extent of pathological destruction, as well as the determination of progression risk in the response to conventional therapy. The criteria that define periodontitis are mostly surrogate endpoints that measure localized tissue destruction. This only allows the quantification of past destruction and hence, limitations on prediction of future disease activity. Additionally, physical measurements are subject to error and variability between clinicians (Goodson et al., 1984). In accordance to the dynamic nature of periodontitis, the practical prognostic systems are fairly arbitrary (McGuire and Nunn, 1996; Kwok and Caton, 2007).

As the specialty of periodontics evolved over the past centuries, endosseous implants entered as reasonable alternatives in clinical reality. However, novel approaches come with *de novo* challenges. Periimplantitis is a pathological condition occurring in tissue around dental implants (Schwarz et al., 2018). Similar to periodontitis, periimplantitis results in inflammation of the surrounding structures and subsequent peri-implant tissue destruction.

Whether we are evaluating either teeth or implants, clinicians still find themselves challenged to determine which host factors are actually destructive, and which may be protective. The most desirable goal in health care delivery is to accurately monitor disease onset, progression and treatment outcomes.

### Inter-Relationship With Other Oral Diseases

The human microbiota includes thousands of diverse species with about 700 microbial genus members (MacGregor et al., 2005; Arweiler and Netuschil, 2016). The oral ecology with its boundless diversity (of oral microbiota) emphasizes the persistent symbiosis of microorganisms in the state of health. Within the oral cavity, hard and soft tissue surfaces are accessible to the cross-linking interaction of microorganisms and permeability of pathogenic etiologies. Disturbance of this equilibrium leads to the most prevalent oral diseases globally which are dental caries, periodontal disease and cancers of the lips and oral cavity (Frencken et al., 2017; Peres et al., 2019). Commonalities of these prevalent diseases are the patient profile and lifestyle habits (e.g., smoking, diet, stress, etc.). Poor oral health and infrequent dental visits have been shown to place patients at higher risk for developing head and neck cancers (Ahrens et al., 2014). Periodontal disease increases the risk of premalignant mucocutaneous diseases such as *oral leukoplakia* by up to five fold (Meisel et al., 2012). Contrastingly, loss of periodontal tissues may be of diagnostic relevance, as progression due to exacerbation of mucocutaneous diseases that can impair oral hygiene care by patients (Jimenez et al., 2002). Oro-mucosal diseases are associated with painful erosions and ulceration of the soft tissues lining the oral cavity. In order to avoid further discomfort, home care is hindered resulting in the accumulation of bacterial plaque biofilms and eventually in the loss of the mucosal tissues including those tooth-supporting periodontal tissues (Rai et al., 2016). Studies have confirmed the significance of periodontal pathogens and tooth loss as diagnostic markers for oral and head-and-neck cancers. (Mager et al., 2005; Zeng et al., 2013b).

In the chronic condition of periodontitis, the ramifications of long-standing inflammation can impact the oral microflora and leading to microbial dysbiosis. Like in periodontitis, the primary etiology of carious lesions is a microbiological infection combined with additional multifactorial environmental drivers (Kornman et al., 2000; Pitts et al., 2017). Yet, the clinical manifestations and patient symptoms of these two diseases differ significantly. Progression of carious lesions stimulates nerves within hard tooth structures, with a possible influence on the pulpal status–that can elicit pain. On the other hand, periodontitis tends to have a more silent onset with slower rate of tissue destruction and hence often more difficult to diagnose early and predict progression.

### Interrelationship With Systemic Conditions

Today’s appreciation of periodontology is manifold, characterized by a relationship with systemic health. Evidence supporting the link between periodontal disease and systemic conditions is accumulating in the literature (Papageorgiou et al., 2017; Heikkila et al., 2018; Romandini et al., 2018; Sanz et al., 2018; Sen et al., 2018) (Table 1). This relationship is mainly attributed to the constant inflammatory state driven by the presence of virulent oral pathogens. Studies confirmed elevated levels of inflammatory markers in patients with periodontitis when compared to healthy controls (Buhlin et al., 2009). This chronic low-grade inflammation gradually and silently contributes to the aggregate systemic inflammatory burden. Chronic inflammation has been known for its close association with adverse cardiovascular events (Ridker and Silvertown, 2008) as well as the formation of thrombi (Mukhopadhyay et al., 2019).

**TABLE 1:** Key Publications on associations of periodontal disease and systemic conditions based on meta-analyses.

Systemic condition	Meta-Analysis	
	Based on studies exploring biomarkers	Based on epidemiological studies
	Reference	Reference
Diabetes mellitus	(Atieh et al., 2014; Artese et al., 2015; Garde et al., 2019; Baeza et al., 2020)	(Darre et al., 2008; Engebretson and Kocher, 2013; Faggion Jr. et al., 2016; Ziukaite et al., 2018; Jain et al., 2019)
Cardiovascular disease	(Mustapha et al., 2007; Paraskevas et al., 2008; Freitas et al., 2012; Teeuw et al., 2014; Munz et al., 2018; Roca-Millan et al., 2018; Joshi et al., 2019; Munoz Aguilera et al., 2020)	(Bahekar et al., 2007; Blaizot et al., 2009; Almeida et al., 2018)
Rheumatoid arthritis	(Han and Reynolds, 2012; Kaur et al., 2014; Bender et al., 2017; Calderaro et al., 2017; Eskandari-Nasab et al., 2017; Hussain et al., 2020)	(Tang et al., 2017; Qiao et al., 2020)
Obesity	(Akram et al., 2016 *)*	(Papageorgiou et al., 2015 *)*
Irritable bowel disease	*	(Eskandari-Nasab et al., 2017; Papageorgiou et al., 2017; Lauritano et al., 2019; She et al., 2020)
Osteoporosis	*	(Penoni et al., 2017; Xu et al., 2020)
Oral cancer	(Wang et al., 2018; Zhang et al., 2019)	(Zeng et al., 2013a; Yao et al., 2014; Ye et al., 2016)

*, No Meta-Analysis identified since 2007.

Additionally, periodontitis has been associated as a potential risk for increased morbidity for systemic conditions including diabetes mellitus, rheumatoid arthritis, obesity, osteoporosis and adverse pregnancy events (Bartold and Lopez-Oliva, 2000; Papapanou, 2015; Penoni et al., 2017; Sanz et al., 2018; Kang et al., 2019). The bidirectional relationship between diabetes mellitus and periodontitis has been extensively investigated and demonstrates that control of one disease positively impacts the other (Sima and Glogauer, 2013). A recent preclinical study reported the potential effect of periodontal inflammation on the pathogenesis of Irritable Bowel disease. The translocation of oral pathogens during an oral inflammatory state to the intestines may lead to the disruption of the colonization resistance of gut-resident microbiota (Kitamoto et al., 2020).

Although periodontitis primarily involves local destruction of tissues in the oral cavity, its management requires a whole-body, long-term strategy tailored to each individual’s profile. The comorbidity between periodontitis and systemic diseases raises the importance of reducing the cumulative inflammatory burden by pursuing early diagnosis and treatment from a dental point of view (Slots, 2000).

## Prediction Models and Biomarker Selection

### Salivary and Crevicular Fluid Diagnostics

A biomarker *is a defined characteristic that is measured as an indicator of normal biological processes, pathogenic processes or response to an exposure or intervention* (Cagney et al., 2018). There is abundant evidence demonstrating that certain inflammatory biomarkers are elevated for years prior to resulting into clinically significant consequences (Ridker and Silvertown, 2008). These same biomarkers are highly predictive for disease onset when assessed timely and accurately. Oral fluids offer a non-invasive opportunity to weigh risks, predict disease initiation, refine diagnosis and stratify treatment modalities (Giannobile, 2012; Zhang et al., 2016).

**FIGURE 1:**
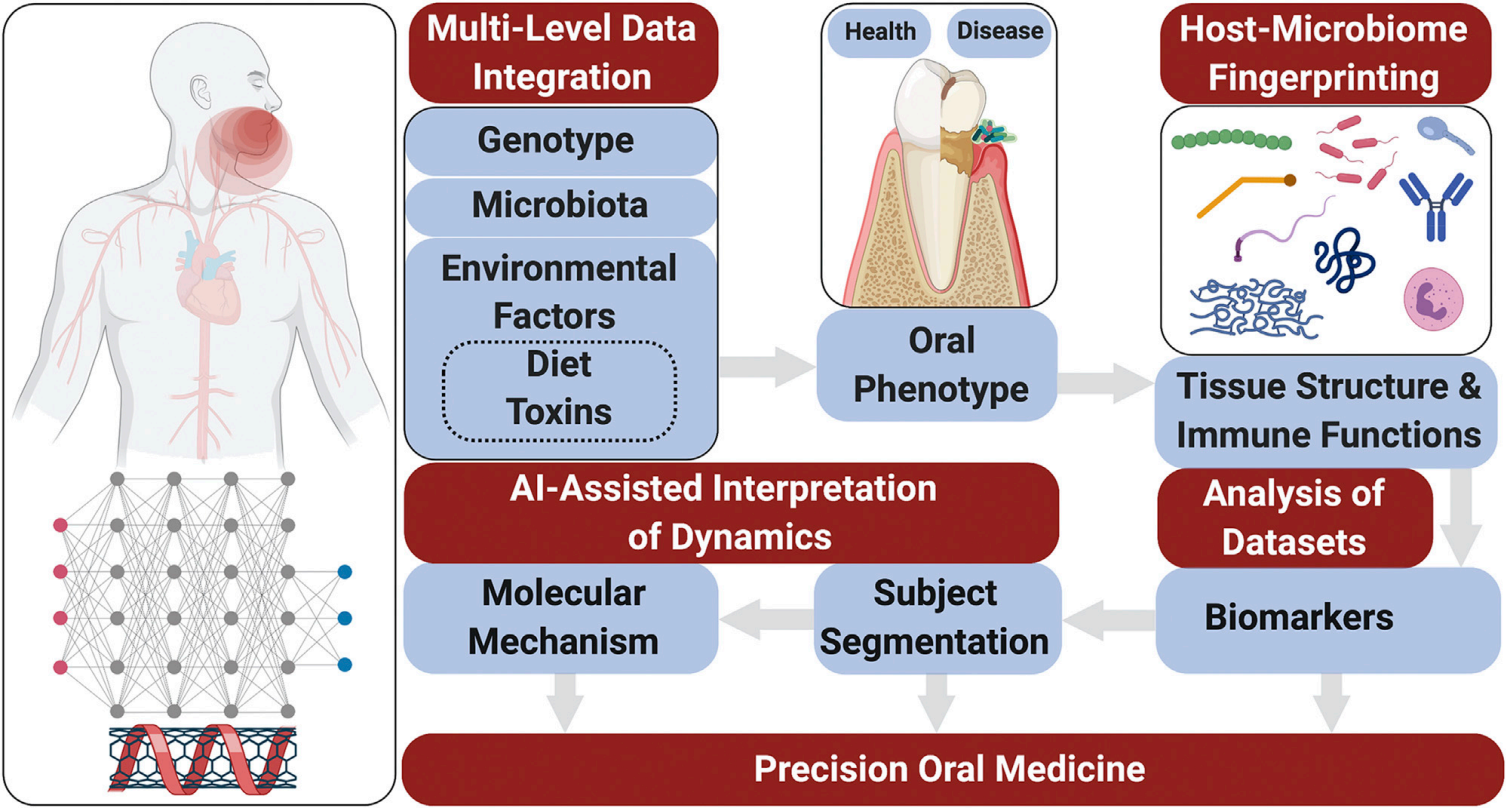
The systems approach to advance precision oral medicine. Schematic diagram illustrating integration of modifiable and nonmodifiable risk factors, and immune-metabolic signatures for biomarker identification, patient stratification, and understanding of molecular mechanisms for oral diseases. The oral soft and hard tissue phenotype is determined by genetic, environmental, and microbial factors. The phenotype is reflected by tissue integrity and immune functions that control the pathogenicity of the oral biofilms. Integrative analysis of data sets (e.g., genomics, proteomics, lipidomics) are used to dissect the changes in tissue structure and immune functions in different subjects and thereby identify biomarkers associated with specific phenotypes. Subject segmentation is necessary for improved clinical trial design and precise treatment approaches. It can also serve as a basis for analysis of dynamic responses to treatment strategies and the identification of molecular mechanisms underlying different phenotypes. Bioinformatic analyses using AI on comprehensive ‘omics data sets to interpret network dynamics across omics layers help develop precise and personalized treatment schemes for oral and associated systemic conditions.

Risk prediction models are generated using microbiological elements, salivary protein biomarkers as well as on a genetic/epigenetic information (Figure 1). The media to obtain these indicators can be derived from human saliva or gingival crevicular fluid (GCF). Numerous studies were carried to analyze the relationships between saliva composition and oral diseases including periodontitis (Brinkmann et al., 2011; Giannobile, 2012; Salminen et al., 2014; Kc et al., 2020), carious lesions (Teng et al., 2015), and oral cancer (Wang et al., 2015; Zhang et al., 2016).

Dating back to 2008, the term “Salivaomics” was introduced to facilitate the research focused on disease diagnosis and monitoring (Wong, 2012). Saliva essentially originates from blood serum and is filtrated by the salivary glands, effectively representing the body’s circulating health or disease markers (Spielmann and Wong, 2011). Saliva is a rich source of proteins related to oral conditions and systemic health. Saliva possesses multiple biological functions such as antibacterial, antiviral, antifungal, wound healing, buffering, tooth mineralization, food digestion, and coating (Vila et al., 2019). In addition to these functions, collection of salivary samples is easy and a non-invasive procedure.

GCF is essentially a serum exudate that accumulates in the gingival sulcus or pocket that is generally rich in biological markers. This site-specific fluid is easily obtainable and generally predictive of periodontal pathogenesis (Zhang et al., 2016). Over the past 2 decades studies have analyzed biomarkers in the GCF and their predictive ability in the periodontitis identification and progression.

#### Microbiological Markers

Since periodontitis is a chronic inflammatory disease associated with microbial dysbiosis and host-driven tissue destruction (Kornman, 2008; Darveau, 2010), it is recognized that a single oral pathogen would not be sufficient to induce the most common forms of periodontitis. However, historically there were several studies focused on small subgroups of patients with rapidly progressing bone destruction occurring around certain teeth (molars and incisors), called localized aggressive periodontitis, which was found to associate with a specific bacterium, named *Aggregatibacter actimomycetemcomitans* (*Aa*). This specific pathogen was once used as a strong diagnostic to monitor treatment outcome (Socransky and Haffajee, 1992) as there were clinical studies showing that sites with residual infection of *Aa* resulted in further clinical attachment loss around teeth (Christersson et al., 1985; Fine et al., 2013). Systemic antibiotic therapy (tetracycline or metronidazole) was indicated to target this pathogen (Slots and Rosling, 1983; Kornman and Robertson, 1985). More recently, the focus has shifted to investigate a separate group of highly virulent bacteria that were identified under the deep periodontal pockets in patients with chronic periodontitis in the general population. They are the “red complex bacteria”(Socransky et al., 1998) or the keystone pathogens (Hajishengallis et al., 2012).

Those red complex and keystone pathogens have been implicated in many studies that showed their association and predictive value for periodontitis. In a 12-months longitudinal study using subgingival plaque with real-time PCR to predict periodontitis progression, red complex pathogens [including *Porphyromonas gingivalis* (*Pg*), *Tannerella forsythensis* (*Tf*) and *Treponema denticola* (*Td*) in conjunction with *E. corrodens* (*Er*), *F. nucleatum* (*Fn*) and *P. intermedia (Pi)*] equally contributed to strong sensitivity and specificity values (Kinney et al., 2014). Additionally, *P. gingivalis* and *T. denticola* in saliva, together with elevated levels of MMP-8 can also be a strong indicator for severe periodontitis (Gursoy et al., 2018; Nascimento et al., 2019; Sorsa et al., 2020). However, at the initial periodontitis lesion, the red complex cannot accurately predict a robust inflammatory response for gingivitis. In a human experimental gingivitis study utilizing checkboard DNA-DNA hybridization, it was found that the orange complex, especially *Fusobacterium nucleatum (Fn)*, predisposes patients to a more pronounced inflammation (Lee et al., 2012). *F. nucleatum* is an early colonizer that bridges the pathogens and thus identified as an emerging opportunistic pathogen that plays a critical role in the dysbiosis of microbiome (Hajishengallis, 2015). However, metatranscriptomic analyses of the human oral microbiome found that *F. nucleatum* upregulates all genes involved inL-lysine metabolism to short chain fatty acid butyrate, which can establish a favorable growth environment for disease-associated communities but is also a beneficial regulator of host-microbiome balance in the gut, and potentially in the oral cavity (Chang et al., 2014; Jorth et al., 2014; Schulthess et al., 2019). The concept of dysbiosis and the recognition of host-microbe interaction mandates the field to not only consider specific oral pathogens but also several host biomarkers including receptors for microbiome metabolites, lipid mediators of inflammation, chemokines, cytokines, enzymes and other proteins. Combining clinical measures, pathogen levels, and cytokines like interleukin (IL)-1β, osteoprotegerin (OPG) and matrix metalloproteinases (MMP)-8 can provide up to 74% sensitivity for predicting periodontitis progression (Kinney et al., 2014).

With the growing use of dental implants, particularly in patients with tooth loss due to periodontitis, peri-implantitis and its increasing prevalence has gained tremendous attention to elucidate the pathogenesis and develop predictable therapeutic approaches. *T. denticola* was found associated with peri-implantitis, and combined with the levels of IL-1β, vascular endothelial growth (VEGF) and tissue inhibitors of metalloproteinases (TIMP)-2 in the peri-implant crevicular fluid (PICF), it enhances the diagnostic accuracy for disease activity (Wang et al., 2016). In a 6 months, prospective interventional clinical trial, lower post-treatment pathogen levels in a panel of oral bacteria, together with >30% reduction of IL-1β, VEGF, and IL-6 in PICF had a 77% sensitivity to predict a favorable treatment outcome (Renvert et al., 2017). More recently, using next generation sequencing technologies, previously unculturable potential pathogens were identified in periodontitis and peri-implantitis lesions, and the level of dysbiosis highly correlated with disease severity (Kroger et al., 2018; Shi et al., 2018).

The methodological advancements in high-throughput sequencing and bioinformatics have provided unprecedented resolution in profiling the oral microbiome and host-microbiome interfaces. Using molecular methods, we have significantly expanded the number of bacterial species (more than two hundred) identified in oral microbiome. However, the field is calling for functional investigation of the bacteria within the biofilm microenvironment and of the associated host oral immune functions to elucidate the ultimate impact of host-microbe interaction on dysbiosis and tissue destruction (Wade, 2011; Krishnan et al., 2017). For example, patients with localized aggressive periodontitis may have selective impaired phagocyte functions (especially for *F. nucleatum*) resulting in overgrowth of opportunistic pathogens relative to commensal bacteria, which represents a potential mechanism for “host-mediated dysbiosis” of the microbiome (Wang et al., 2015). Ultimately, understanding the host-microbe interactions and the influence of dysbiosis in each individual holds the key for future diagnostics and therapeutics.

#### Protein Biomarkers

Precision medicine demands a systems wide approach to integrate multi-level data in search for accurate and reliable biomarkers of disease activity, and novel therapeutic targets to improve the management of chronic conditions in all fields of medicine (Giannobile et al., 2013). Protein biomarkers pave the way to individualized prevention, diagnosis and treatment. When considering the periodontal pathogenic processes, periodontitis can be generally divided into three phases: inflammation, connective tissue degradation, and bone resorption. During each phase of the disease activity, specific biomarkers have been identified to provide a general sense of what stage of pathologic breakdown the patient is currently residing (Korte and Kinney, 2016) (Table 2). Numerous studies were carried out to analyze the relationships between oral diseases and saliva composition. The most recent systematic review reported macrophage inflammatory protein-1 alpha, IL-1β, IL-6, and MMP-8 as promising diagnostic biomarkers (Kc et al., 2020).

**TABLE 2:** Oral-based biomarkers with major effector functions associated with oral and systemic diseases.

Type of Biomarker		Major effector functions	References
Inflammation	IL-1β	Potent proinflammatory stimulator	(Kornman et al., 1997; Akdis et al., 2011)
		Potent effects on cell proliferation, differentiation and function of many innate and specific immunocompetent cells	
		Strong correlation with periodontal disease progression	
	IL-6	Regulator of T- and B-cell growth	(Akdis et al., 2011; Lee et al., 2012)
		Directs leukocyte trafficking	
		Induces production of acute-phase protein	
		Increase levels of periodontal disease	
	IL-10	Restriction of excessive inflammatory responses	(Ouyang and O’Garra, 2019)
		Upregulation of innate immunity and promotion of tissue repair mechanisms	
	IL-8	Recruitment and activation of neutrophils	(Lee et al., 2012)
		Attracts NK cells, T cells and basophils
	TNF-α	Macrophage activation	(Erdemir et al., 2004; Akdis et al., 2011)
		Inducing apoptosis of epithelial cells in the mucosa	
		Regulates MHC class I and II protein and antigen presentation expression	
		Stimulates gingival fibroblasts to produce collagenase	
	CRP	Increases rapidly in response to trauma, inflammation and infection	(Freitas et al., 2012)
		Activates the complement pathway, apoptosis, phagocytosis, nitric oxide (NO) release, production of cytokines	
	IFN-γ	Key cytokine in bridging innate and adaptive immune system	(Akdis et al., 2011)
		Regulate MHC I and II class protein expression	
		Inhibition of cells growth primarily by increasing levels of cyclin-dependent kinase inhibitors	
		Proapoptotic affects	
	PGE2	Lipid mediator that regulates activation, maturation and cytokine secretion of several immune cells	(Agard et al., 2013)
		Induced during bacterial pathogenesis	
Tissue destruction	MMP-8	Degradation of interstitial collagens	(Kinney et al., 2007)
		Prevalent host proteinase in periodontal disease	
	MMP-9	Proteolytic degradation of extracellular matrix proteins	(Cui et al., 2017)
		Mediator of tissue destruction and immune responses in periodontal disease	
	MMP-13	Expressed by epithelial cells during prolonged inflammation	(Offenbacher et al., 2010)
		Efficiently degrading type II collagen	
	TIMP	Naturally occurring MMP inhibitor that bind MMPs in a 1:1 stoichiometry	(Pietruska et al., 2009; Cui et al., 2017)
		Decreased levels after periodontal treatment	
	Cathepsin-B	Degrades extracellular components, type IV collagen, laminin and fibronectin	(Buck et al., 1992)
Bone remodeling	OPG	Decoy receptor for RANKL	(Bostanci et al., 2007)
		Inhibits osteoclast formation	
	RANKL	Stimulates RANK on the surface of stem cells to form osteoclasts	(Bostanci et al., 2007)
		Regulation of bone destruction	
	ICTP	Pyridinoline cross-links with high specificity for bone (compared to histidine cross-links for soft tissue and skin)	(Giannobile, 1999; Al-Shammari et al., 2001)
		Osteoclastic bone resorption initiates the release of cross-linked immunoreactive telopeptides	
	Calprotectin	Antimicrobial and antifungal activities (improving resistance to *P. gingivalis*)	(Kinney et al., 2007)
		Inhibits immunoglobulin production	
		Neutrophil recruitment and production	
	Osteonectin	Affinity to collagen and hydroxyapatite leading to tissue mineralization	(McCauley and Nohutcu, 2002)
		Key role in remodeling and repair	
	Osteocalcin	High concentration during bone turnover	(Giannobile et al., 1995)
	Osteopontin	Highly concentrated at sites where osteoclasts are attached to the underlying mineral surface	(McCauley and Nohutcu, 2002; Kinney et al., 2007)
		Holds a dual function in bone maturation and mineralization as well as bone resorption	
		Highly glycosylated extracellular matrix protein with levels in active sites of bone metabolism	

IL, Interleukin; NK cells, Natural killer cells; TNF, Tumor necrosis factor; CRP, C-reactive protein; MHC, Major histocompatibility complex; IFN, Interferon; PGE, Prostaglandin E; MMP, matrix metalloproteinases; TIMP, Tissue inhibitor of metalloproteinases; OPG, Osteoprotegerin; RANKL, Receptor activator of nuclear factor kappa-B ligand; ICTP, C-telopeptide pyridinoline cross-links.

The pro-inflammatory cytokines IL-1β and tumor necrosis factor alpha (TNF)-α were frontrunner targets in the quest for molecular biomarkers of periodontitis activity (Page and Kornman, 2000). Decreases in IL-1β and TNF-α levels were found to reduce periodontitis progression (Delima et al., 2001). Further, the relationship between inflammatory cell infiltrates and tissue degradation is centered on the production of MMPs: collagenases, gelatinases and the stromelysins. They are synthesized by resident gingival and innate immune cells including epithelial cells, fibroblasts, neutrophils and macrophages. TIMPS control the local extracellular MMP activity in tissue remodeling, allowing a constant balance of reparative and destructive phases in soft tissue matrices (Meikle et al., 1994; Reynolds and Meikle, 2000). In the same sense, inflammatory osteolysis and bone remodeling are regulated by several bone metabolites and immune mediators (Giannobile, 1999; McCauley and Nohutcu, 2002; Kinney et al., 2007). Receptor-activator of NF-κβ ligand (RANKL), a member the TNF-superfamily, is an essential pro-osteoclast factor, which, together with its decoy receptor OPG, is critical for bone resorption-formation coupling. RANKL binds to RANK, which leads to the activation and differentiation of osteoclasts with subsequent bone destruction. In active periodontitis, levels of RANKL are increased and levels of OPG are decreased, therefore RANKL/OPG ratio may serve as a biomarker for periodontitis.

Recent studies have shown that site specific GCF biomarkers can be effective measures of periodontal treatment efficacy [e.g., hypoxia induced factor-1 alpha (HIF-1α)*,* VEGF and TNF-α levels] and hence their ability to surveil therapeutic modalities (Afacan et al., 2020). When evaluating the sensitivity of GCF-based tests, longitudinal studies found that addition of biofilm-associated pathogens may have higher predictive value for periodontitis progression (Kinney et al., 2014).

#### Use of Genetics for Risk Stratification and Prognosis

Page and Kornman suggested that the pathological mechanism of periodontitis, is centered around the host’s responses against microbial challenges, a paradigm that continues to this day (Hajishengallis and Korostoff, 2000; Page and Kornman, 2000). Environmental and genetic risk factors are mediators of the host immune-inflammatory response, and connective tissue and bone metabolism. The susceptibility to periodontitis is fundamentally polygenic in nature and regulated through genes induced by environment interactions (Schaefer, 2000). The heritability of periodontitis has been investigated in familial and twin studies, estimating to be around 30–50% after being adjusted for covariates (Michalowicz et al., 2000; Nibali et al., 2019).

The human genome sequencing and subsequent functional investigations advanced our understanding of genetic regulation, gene by environment interactions and their roles in complex chronic conditions. The current emerging paradigm proposes that specific disease phenotypes can be attributed to the effects of combinations of various genetic risk alleles and their interaction with internal and external factors (Schaefer, 2000). Severe periodontal genotypes may carry susceptibility alleles more frequently. IL-1 is a potent pro-inflammatory cytokine and is involved in the pathogenesis of many human chronic conditions including cardiovascular, metabolic, autoimmune and others (Sims and Smith, 2010). For periodontitis, the IL-1 genotype has a regulatory effect on the local inflammatory processes (Kornman et al., 1997; Offenbacher et al., 2018). IL-1A allele 2 and IL-1B+3953 allele 2 genotypes were recognized as strong indicators for severe periodontitis in an adult populations (Kornman et al., 1997). Carriers of these alleles produce more IL-1 by blood mono-and polymorphonuclear cells, and have a higher IL-1 prevalence in GCF (Rogus et al., 2008; Latella et al., 2009). IL-1 genotypes have been consistently associated with periodontitis within diverse ethnic groups. A high frequency of the IL-1b allele 1 was observed in localized juvenile periodontitis patients in an African-American population (Walker et al., 2000).

Longitudinal studies of IL-1 gene polymorphisms (Cullinan et al., 2001) and the genome-wide association studies (GWAS) shed light on the IL-1 role as a critical risk factor for periodontitis and determinant of the pro-inflammatory host response. These findings support patient stratification based on the IL-1 genotype for tailored measures to prevent periodontitis progression and associated tooth loss (Giannobile et al., 2013).

Epigenetics implies DNA modification that does not change the DNA sequence, but rather influences gene expression and the consequent disease activity (Schmidl et al., 2018). Epigenetic alterations are known to affect transcription and translation of genes through DNA methylation, post-translational modification of histones and/or non-coding RNA (Barros et al., 2000; Larsson et al., 2015). Several studies have found hypomethylation profiles within the interferon (IFN)-γ, IL-6, and TNF-α promoters, which was associated with increased IFN-γ, IL-6, and TNF-α transcription in gingival biopsies from patients with periodontitis (Zhang et al., 2010; Kobayashi et al., 2016). Furthermore, a hypermethylation pattern of the prostaglandin-endoperoxide synthase 2 (PTGS2) promoter was found associated with reduced PTGS2 transcription, consistent with a dampening of cyclooxygenase- expression that had been reported in gingival samples collected from sites with periodontitis (Zhang et al., 2010). One study found that microbial challenges induced changes in DNA methylation affecting the barrier function of oral epithelial cells (Barros et al., 2020).

The impact of risk variants and their interaction on modifying disease susceptibility is currently being investigated (Freitag-Wolf et al., 2019). Complex diseases like periodontitis are significantly influenced through genes by environment interactions. McGuire et al. reported that the IL-1 genotype-positive heavy smoker patients had significantly worse tooth survival rates (McGuire and Nunn, 1999). Parkhill reported that IL-1β, IL-1-Receptor Antagonist and IL-1β genotype combined with smoking in Caucasians are risk factors for early onset periodontitis (Parkhill et al., 2000). Smoking was associated with DNA hypomethylation in the SOCS1 promoter of epithelial cells isolated from saliva of periodontitis subjects (Martinez et al., 2019). Also, DNA hypomethylation of the IL-8 gene promoter in oral epithelial cells from periodontitis subjects was decreased (Oliveira et al., 2009).

Advances in analyzing equipment have recently been developed and the next generation sequencer (NGS) has emerged as a powerful tool. Due to NGS, GWAS have been widely accepted and performed worldwide to elucidate linkages between genetic background and potential susceptibility to diseases.

GWAS for periodontal diseases reported 41 consensus master regulator genes (Sawle et al., 2016) and signals for periodontal complex traits (Offenbacher et al., 2016). More specifically, in patients with chronic periodontal conditions four genes (*NIN, ABHD12B, WHAMM,* and *AP32*) have been identified (Rhodin et al., 2014). Sanger sequencing revealed the association of NOD2 mutations in a Japanese population with aggressive periodontal disease (Sudo et al., 2017).

Due to the diversity in the pathogens, environmental factors and genetics, further studies need to be performed in order to develop their association with periodontitis.

## Diagnostic Devices

### Salivary Proteome and Salivary Genome

The expression *proteome* combines the terms *proteins* and *genes,* and implies the simultaneous study of all proteins in a cell and protein complements of the genome (Parker et al., 2010). Studies in periodontology using proteomic analysis of whole saliva from chronic periodontitis patients were able to show the suppression of several salivary proteins related to protective functions and oral homeostasis (Hartenbach et al., 2020).

The advancement of analyzing equipment such as NGSs and mass spectrometry (MS) allowed the emergence of the new field of “Omics.” Omic technologies analyze genetic or molecular profiles and obtain a complete assessment of the functional activity of biochemical pathways with their structural genetic sequence differences among individuals. (Aardema, 2002). The complementary advances in information technology and sequencing devices in this decade have enabled analysis of vast amounts of Omics information, contributing to better understanding of disease activity to improve our prediction for disease onset and progression (Eicher et al., 2020). In recent years, various investigations were carried out using preclinical animal and translational Omics analyses to elucidate pathological mechanisms (Rhodin et al., 2014; Offenbacher et al., 2016; Sawle et al., 2016; Sudo et al., 2017; Maekawa et al., 2019; Shin et al., 2019). Since Omics probe different cellular layers, they are categorized at DNA, RNA, protein, or metabolite levels (Yugi et al., 2016). Accordingly, current studies encompass genomics/epigenomics, transcriptomics, proteomics and metabolomics. The oral microbiome research has recently moved to a higher level of granularity using these approaches to both host and microbe investigations. For example, transcriptomics allows us to understand the conserved disease-associated gene expression profile in the microbiome for the onset of gingivitis and progression to periodontitis (Yost et al., 2015; Nowicki et al., 2018). Another more general approach is metagenome analysis, with the purpose to identify novel biocatalyst genes to increase our understanding of microbial ecology (Streit and Schmitz, 2004). It enables investigators to screen for activities and functions of interest.

Studies in periodontology using proteomic analysis of whole saliva from chronic periodontitis patients were able to show the suppression of several salivary proteins related to protective functions and oral homeostasis (Hartenbach et al., 2020). In 2019, deep sequencing combined with rapid shotgun analysis for ultra-deep and quantitative saliva proteome characterization identified 5,500 host proteins and more than 2,000 microbial proteins in samples of periodontitis patients, with a strong correlation between dynamics of the oral microbiome and salivary proteins (Grassl et al., 2016).

The “shotgun” proteomics analysis revealed that S100A8 and S100A9 in saliva are potential biomarkers for active periodontitis (Shin et al., 2019). In support of this, several studies have revealed their pathogenic roles in periodontitis progression (Maekawa et al., 2019; Liu et al., 2020). S100A8 and S100A9, two pro-inflammatory calcium-binding S100 proteins acting as damage-associated molecular patterns, were implicated in pathogenesis of rheumatic diseases having strong associations with the disease activity and potential as therapeutic targets (Austermann et al., 2018). S100A9 was also reported to increase IL-6 production and RANKL expression in osteocyte-like cells suggesting potential as osteoimmune biomarker for inflammatory osteolysis (Takagi et al., 2020).

Liquid biopsies are an emerging innovative strategy with reduced invasiveness, especially in the medical field of cancer (Heitzer et al., 2019). So far, biopsy samples are mainly blood and serve for the detection of tumor components including circulating tumor cells, circulating tumor DNA, cell-free RNA, extracellular vesicles, and miRNA. Unlike solid biopsies, liquid biopsy are less invasive and easier to obtain with the purpose of early disease detection (Sierra et al., 2020), prognosis and follow-up (Wan et al., 2017). Recently, attention has also been drawn toward oral fluid-based liquid biopsies for detecting RNA and miRNA in saliva and GCF.

miRNAs are gene regulatory molecules in multicellular organisms and likely influence the output of many protein-coding genes (Bartel, 2004). Over the past decade, research related to microRNAs (miRNA) in periodontitis has accumulated (Kebschull and Papapanou, 2000; Xie et al., 2011; Perri et al., 2012; Stoecklin-Wasmer et al., 2012). To date, 25 miRNAs were reported to be significantly up-regulated and six miRNAs down-regulated in inflamed gingival tissues in periodontal patients (Luan et al., 2017; Luan et al., 2018). Several studies on the expression of miRNAs using various dental tissue cells such as gingival fibroblasts (Sipert et al., 2014; Matsui and Ogata, 2016) and PDL cells (Chen et al., 2015) have been performed (Sehic et al., 2017).

Recently, Saito et al. investigated 600 miRNAs in GCF and found that they had a different profile compared to GCF in healthy subjects (Saito et al., 2017). The expression of miR-1226 in GCF in patients with severe chronic periodontitis was significantly down-regulated (Mico-Martinez et al., 2018) while other groups reported that miRNAs associated with inflammation (e.g., miR-146a and miR-155) were significantly up-regulated in chronic periodontitis with type 2 diabetes. Furthermore, pilot studies have reported increased expression of miR-143-3p (Nisha et al., 2019), miR-381-3p (Fujimori et al., 2019) in chronic periodontitis patients. Interestingly, these microRNAs expressions in GCF were reduced after non-surgical therapy when compared to the level of healthy controls (Radovic et al., 2018).

Due to the scarcity of studies on miRNAs in GCF and saliva (Saito et al., 2017; Atsawasuwan et al., 2018; Mico-Martinez et al., 2018; Radovic et al., 2018; Fujimori et al., 2019; Al-Rawi et al., 2020) further studies are still needed. Wearable sensors in the oral cavity may allow detection of miRNA changes, which may be useful for more detailed diagnosis and tracking of dynamics in periodontitis.

In search for functional and regulatory periodontitis biomarkers small extracellular vesicles (sEV) in saliva related to miRNAs were recently investigated. One study reported that three miRNAs, hsa-miR-140-5p, hsa-miR-146a-5p and hsa-miR-628-5p in sEV, may have potential as diagnostic biomarkers for periodontitis (Han et al., 2020).

Modern matrix-assisted laser desorption ionization time-of-flight (MALDI-TOF) MS can perform quick and accurate proteome analyses to characterize a wide spectrum of samples. A study using MALDI-TOF MS examined the differentially expressed peptide peaks in saliva, GCF, and dental plaque samples and revealed high sensitivity and specificity in diagnosing periodontitis (Antezack et al., 2020).

**FIGURE 2:**
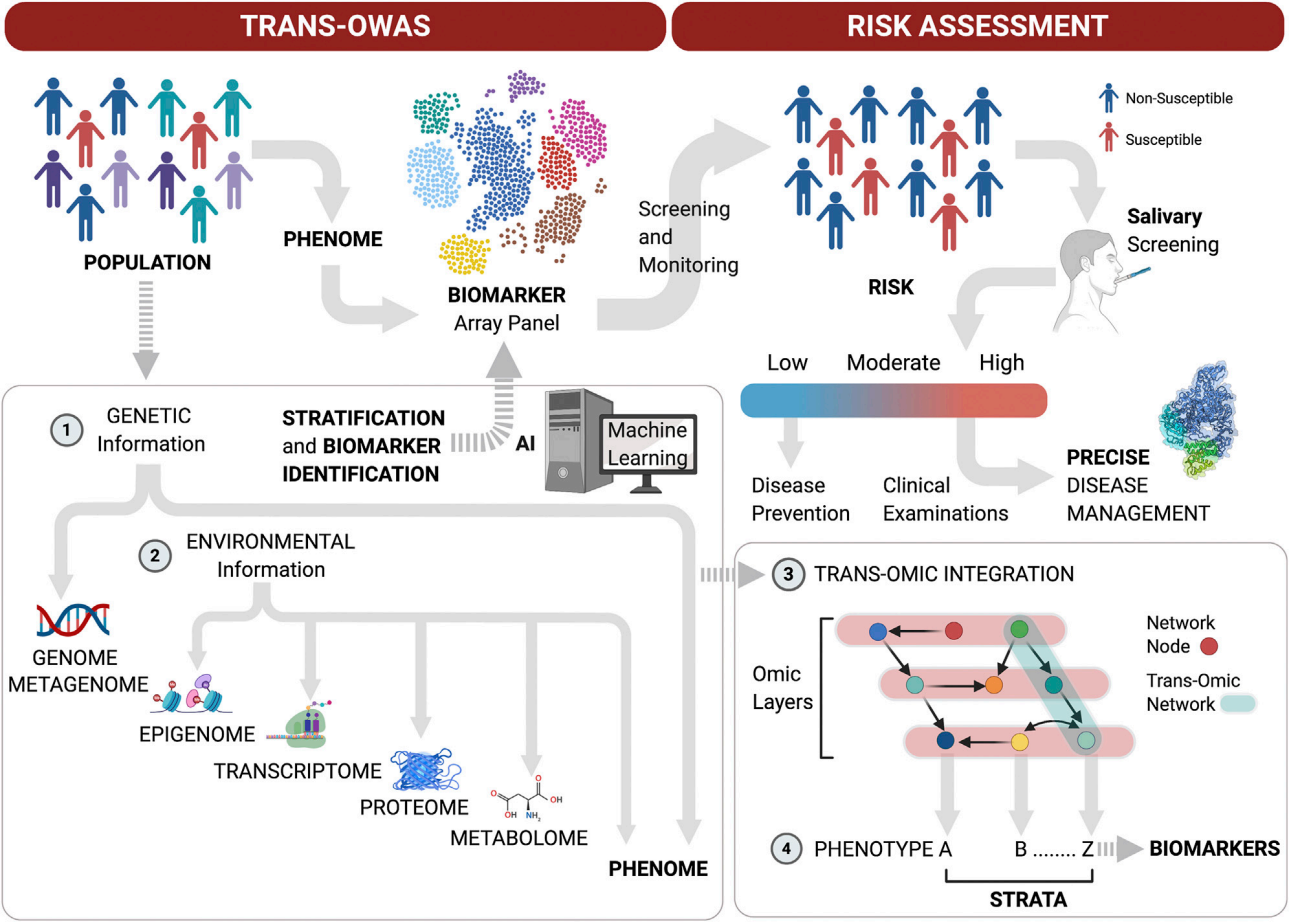
Patient stratification workflow for oral screening and monitoring. Multi-level omics analyses (*Trans*-OWAS) analyses are used to construct the relationships between periodontal phenotypes, which collectively define the phenome, and key biomarkers identified across omics layers. The combination of genetic (1) and environmental information (2) to develop deep learning algorithms from dense population-wide data helps us design periodontal phenotype-specific biomarker panels to more accurately and precisely predict disease progression and response to therapy. This further allows for validation of diagnostic systems based on pathogenesis rather than amount and pattern of tissue destruction. Population-wide *trans*-OWAS integration (3) and deep learning identify and perpetually redefine phenotypes and refine by-phenotype biomarker panel array systems (4). This facilitates patient stratification for high predictive values of tests to determine disease susceptibility. Salivary screening for disease activity biomarkers classifies individuals as being at low, moderate or high risk for disease onset or progression. This allows for timely disease assessment and precise management by combining clinical examinations with further targeted salivary tests and pharmacogenomic analyses.

Taken together, these technological advances in Omics performance and the need for a systems approach to achieve the goal of precision medicine highlight the next Frontier in multi-level Omics studies on large populations to define periodontitis phenome and refine biomarker arrays with high predictive values for disease progression. Moreover, multi-level omics analyses (*Trans*-OWAS) emerge to elucidate more comprehensive mechanisms in diseases to ultimately define disease phenotypes based on pathogenesis rather than amount and pattern of tissue destruction (Yugi et al., 2016). *Trans*-OWAS analysis is reconstructing a global biochemical network by connecting multi-omic layers. Further technologies, combining multi-omics with deep learning will help develop diagnostic systems using various biomarkers, clinical parameters and demographic data to create databases that perpetually refine the phenome (Figure 2).

### Biosensors and Microelectro-Mechanical Systems

The most reliable and common methods for detecting protein and gene expression are conventional techniques such as ELISA and quantitative PCR (qPCR), respectively. These analyses achieve both high sensitivity and high specificity. However, qPCR analysis requires physical manpower, prolonged reaction times, expensive equipment and significant training (Zhu et al., 2020).

In recent years, the accelerated progress in biotechnologies led to the development of biosensors and microelectro-mechanical systems (MEMS). Biosensors recognize the analytes that are sent to reader devices via electrical signals (Kim et al., 2019). MEMS essentially miniaturized the system of biosensors using microfluidics, microdevices and micro total analysis systems with a reduction of the reagents and reaction time (Seker et al., 2011). These technologies enable the rapid detection of analytes with greater sensitivity. Currently, toxins can also be detected using biosensors (Chauhan et al., 2016). Recent advancements that occurred in the field are significant. However, overall commercialization and clinical translation seems to be modest (Kaisti, 2017).

Lab-on-a-chip (LOC) is a subset of MEMS integrating laboratory function on an integrated chip. LOC can provide *in vitro* diagnostic results ad-hoc. After the recognition of the specific analyte, the information is transduced to an integrated chip (Nguyen et al., 2018). In 2020, a new polymeric LOC based on a microfluidic capillary flow assay with on-chip dried reagents was created to detect unbound cortisol in saliva. Increased cortisol levels were reported in mentally-challenged patients (Heinze et al., 2016), thus monitoring cortisol level in saliva can be a valuable predictor for early detection of psychological disorders.

Another technique known as integrated two-dimensional paper network implements an automated multi-step processing for viral detection, enabling us to identify the presence of viruses within minutes. Influenza virus can be identified by surface glycoproteins and hemagglutinin. This technology has already been applied to other viruses such as norovirus, rotavirus, adenovirus, influenza virus, and respiratory syncytial virus (Zhu et al., 2020).

**FIGURE 3:**
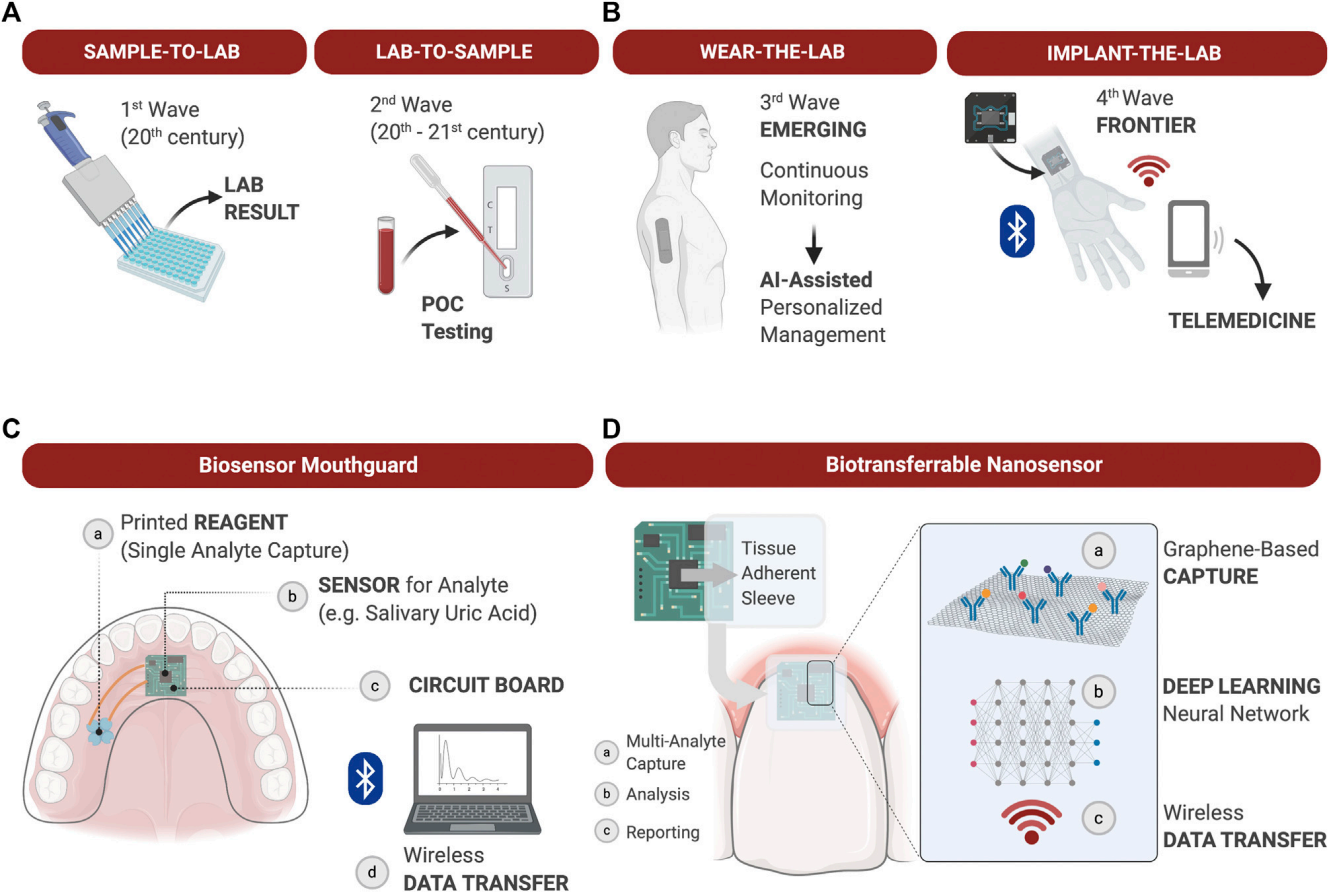
The evolution of diagnostic devices and wearable lab-on-a-chip’s (LOC) for precision medicine applications. **(A)**: The most common diagnostic approaches to measuring soluble biomarkers are “sample-to-lab” and “lab-to-sample,” i.e., samples are either collected from patients, transferred to the lab and analyzed, or tests are delivered directly at the point-of-care for rapid actionable results in the clinic. Technological advancements of the 21st century allow for the development of LOC analyzers to gather diagnostic information chairside in real time. **(B)** The emerging integration of wearable LOC’s in health care allows for continuous monitoring of physiological and pathological processes, and provide dense individual-level data for Artificial Intelligence (AI)-assisted personalized management. The next Frontier in LOC development may be the fabrication of biocompatible implantable sensors for continuous measurement of soluble biomarkers difficult to measure through the skin. Such advancements will expand diagnostic capabilities, at-home care and telemedicine; **(C)**: Example of a wearable biosensor integrated into a mouthguard to capture a single analyte in saliva over time and transduce the signal via Wi-Fi for analysis; **(D)**: Example of a graphene-based nanosensor adhered to the tooth surface and marginal gingiva to capture and quantify multiple analytes over time. Data is processed onboard and deep learning algorithms applied to establish personal physiological thresholds and out of personal norm trends. Wirelessly transferred output data supports clinical decisions during in-office or teledentistry appointments.

Various field-effect transistor sensors can enable detection of ions, molecules, oligonucleotides and proteins in a fast response time (Kaisti, 2017). Furthermore, biosensors using wireless graphene nanosensors are able to detect oral microorganisms at a single cell level on hard tissue surfaces like tooth enamel (Mannoor et al., 2012). Mannoor et al., 2012 confirmed that biosensors placed on a tooth were capable of remote monitoring and detection of bacteria in saliva. Additionally, biosensors on mouthguards have been reported to analyze metabolites (Kim et al., 2014) and the uric acid (Kim et al., 2015) in saliva. This device could detect analytes with high sensitivity and stability, and monitor them in a real-time manner. Heikenfeld et al. (Heikenfeld et al., 2019) reported recent trends to wear the lab, and in the future the implantation of the lab could follow. In oral environments, there are many opportunities to place the LOC technology in association with restorations of teeth or dental implants, or simply wear the LOC embedded into a mouthguard (Figure 3).

Focus should be directed to discover oral locations in order to monitor patient health conditions, systemic disease, and detect viral and bacterial infections.

Various technologies emerge constantly and develop rapidly. Machine learning using artificial intelligence (AI) accelerates the development of identification of biomarkers for application in dental medicine (Bianchi et al., 2020). As of this moment, technologies have developed to a point where we have achieved a reduction in size of sensors, providing multiple salivary analytes simultaneously and improvements in the sensitivity and specificity of testing. Ongoing developments and innovative approaches will help in the detection of the risk of oral disease, disease onset, and monitoring of oral and systemic conditions.

## COVID-19 Point-of-Care Testing in Oral Health Care Settings

**FIGURE 4:**
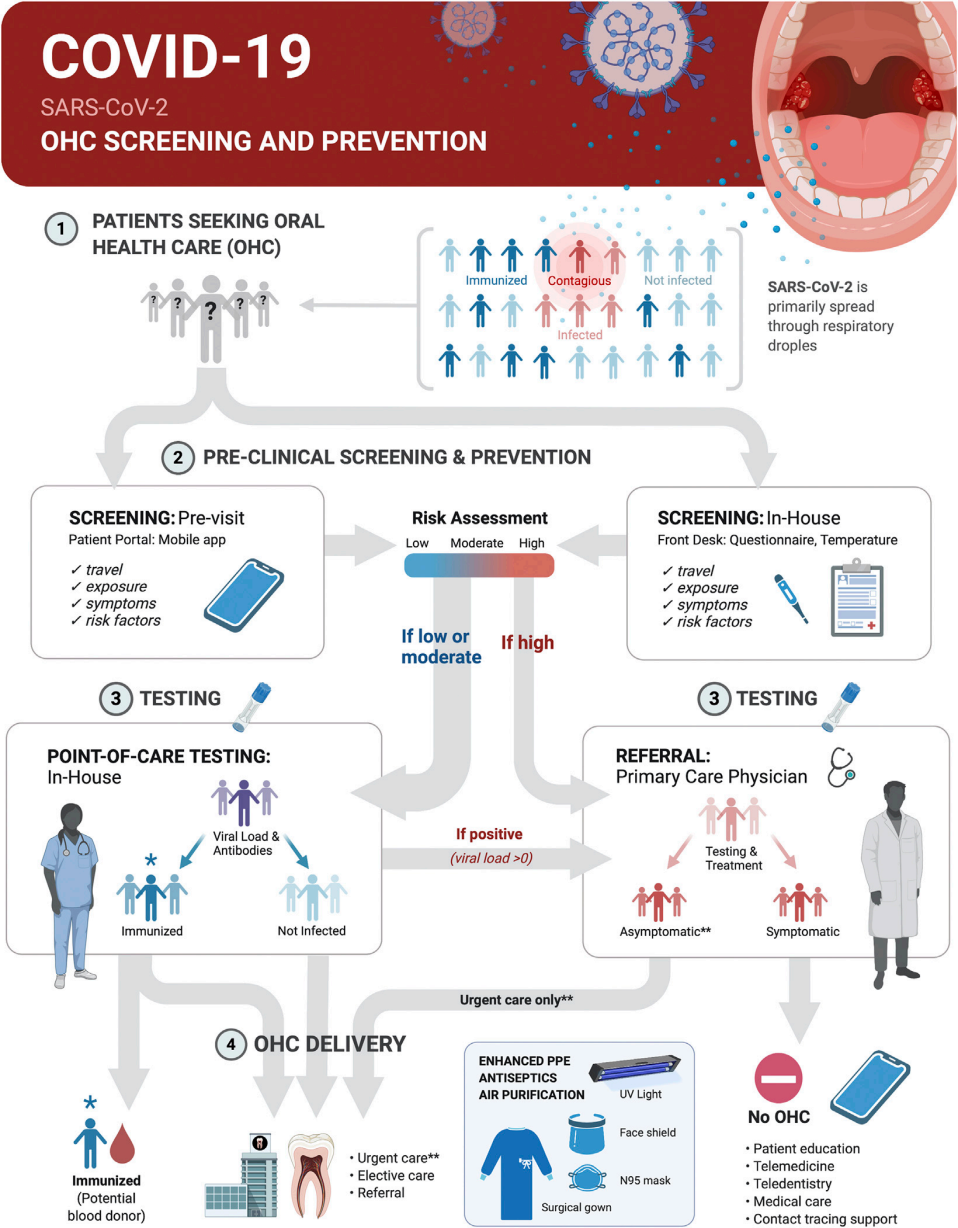
Screening and SARS-CoV-2 point-of-care (POC) Testing for Oral health care (OHC) Decision Making and Serosurveillance. Screening and testing can provide actionable results at POC. COVID-19 screening and prevention protocols in OHC settings should be implemented within the clinical decision trees on delivery of elective or urgent care. Pre-appointment screenings via patient portals or mobile phone apps supplemented with in-house measurement of body temperature and POC testing can be the basis for safe practices and rational use of advanced personal protective equipment. This protocol first establishes who is at low-to-moderate risk (<65 years old, no known risk factors for severe disease outcome, no known exposure to individuals with active disease or recent travel to and from locations with outbreaks, no symptoms and temperature <100°F/37.8°C) vs. high-risk (>65 years old, existing risk factors for severe disease outcome, known exposure to individuals with active disease or travel to and from outbreak locations in the past 14 days, or present symptoms and body temperature >100°F/37.8°C). It then determines by testing if a patient was likely never infected, was previously infected and is immunized or is currently infected with SARS-CoV-2. Non-infected and immunized patients will benefit from both urgent and elective care. Infected asymptomatic should benefit from urgent care only with appropriate prevention measures. All treatments should be deferred for symptomatic infected individuals until recovery. Recovered COVID-19 patients with undetectable virus should benefit from urgent and elective care. Referral of positive cases to primary care physicians, and of immunized, but virus negative patients to blood donation, and contact tracing support will contribute to early management and control of disease spread.

Oral health care (OHC) practices can contribute to broad-based COVID-19 prevention and management measures through screening, point-of-care (POC) diagnostic tests and appropriate referral of patients in these settings (Figure 4). The integration of POC testing platforms for nucleic acids and antibodies into OHC workflows will allow for continuity of care, rational use of advanced personal protective equipment, reduction in transmission at community level, serosurveillance and contact tracing support during pandemics with pathogens transmitted through respiratory droplets, including SARS-CoV-2.

SARS-CoV-2 is detected in saliva of infected individuals (Wyllie et al., 2020; Azzi et al., 2020; Iwasaki et al., 2020; Sabino-Silva et al., 2020; To et al., 2020a; To et al., 2020b). Both saliva and posterior oropharyngeal swab sampling are less invasive, more acceptable to patients, and reduce exposure risks for healthcare workers compared to nasopharyngeal swab sampling. Therefore, salivary diagnostics can provide a convenient and cost-effective POC platform for fast detection of SARS-CoV-2 RNA. Further, using reliable self-collection devices can facilitate direct-to-patient diagnostic testing. The FDA has authorized specific SARS-CoV-2 testing on saliva samples collected at home using a designated self-collection kit (FDA, 2020). The feasibility and reliability of POC testing in OHC settings depends on simplicity and accuracy of the assay, availability and training of personnel to carry out the tests, and efficient integration without significant interruption of care.

New serological tests are being developed as IgM, IgG or combined lateral flow or ELISA assays to measure antibodies against SARS-CoV-2. They are intended for the qualitative detection and differentiation of IgM (developed early during infection) and IgG (developed late during infection) antibodies to SARS-CoV-2 in serum, plasma or venipuncture whole blood specimens from patients suspected of COVID-19 by a healthcare provider. The validation of such assays on fingerpick blood samples supports the feasibility of testing for anti-SARS-CoV-2 antibodies at the POC, including in OHC settings. It is important to note that: 1) assays to certify an individual’s immunity need to be correlated with protection and have near-perfect specificity when seroprevalence is low e.g., 5% or less; 2) assays to ascertain population-level exposure need well defined sensitivity and specificity for the target population, allowing for adjustment of seroprevalence estimates (Bryant et al., 2020). Further, identification of viral RNA via PCR-free isothermal reaction allows for SARS-CoV-2 identification in saliva samples, but large studies are needed to establish assay accuracy and precision. To achieve precise estimates of disease burden, optimal thresholds for sensitivity/specificity should be set depending on local prevalence and intended use of the assay, prioritizing specificity at the expense of sensitivity in low-prevalence settings, and the opposite in high-prevalence settings.

## Future Perspectives

This review presented a general overview of innovation in point-of care in the periodontal field, however emerging research regarding this topic is ongoing and will allow for future systemic analyses as more data becomes available. There have been many advancements in the development of rapid, chairside POC diagnostics in the biomedical diagnostics arena for the timely assessments of disease diagnosis. With the strong needs within the infectious diseases field for non-invasive and rapid assessment, the use of LOC microfluidics and biosensors offers new avenues in oral disease identification and risk assessment. While the detection of biomarkers of disease in periodontology remains overall limited, innovations in biomedical engineering have provided strong potential in dental medicine. The coupling of computational approaches (AI, machine learning and deep learning) will exploit clinical, biological and other omics data elements based on large data sets for the better stratification of patients. The multi-analyte technologies and use of these approaches will undoubtedly lead to more accurate and comprehensive determinants of other polygenic diseases beyond periodontitis.

## Author Contributions

All of the authors were involved in the preparation of the manuscript and give consent to the integrity of the contents for consideration of publication.

## Funding

The work was supported by the Osteology Foundation.

## Conflict of Interest

The authors declare that the research was conducted in the absence of any commercial or financial relationships that could be construed as a potential conflict of interest.
